# The misdiagnosis of prolonged disorders of consciousness by a clinical consensus compared with repeated coma-recovery scale-revised assessment

**DOI:** 10.1186/s12883-020-01924-9

**Published:** 2020-09-12

**Authors:** Jing Wang, Xiaohua Hu, Zhouyao Hu, Ziwei Sun, Steven Laureys, Haibo Di

**Affiliations:** 1 International Unresponsive Wakefulness Syndrome and Consciousness Science Institute, Hangzhou Normal University, Hangzhou, 310036 China; 2 Rehabilitation Center for Brain Damage, Wujing Hospital of Hangzhou City, Hangzhou, China; 3 Coma Science Group, GIGA Consciousness, University and University Hospital of Liège, Liège, Belgium

**Keywords:** Coma-recovery scale-revised, Disorders of consciousness, Unresponsive wakefulness syndrome, Minimally conscious state, Misdiagnosis

## Abstract

**Background:**

Previous studies have shown that a single Coma-Recovery Scale-Revision (CRS-R) assessment can identify high rates of misdiagnosis by clinical consensus. The aim of this study was to investigate the proportion of misdiagnosis by clinical consensus compared to repeated behavior-scale assessments in patients with prolonged disorders of consciousness (DOC).

**Methods:**

Patients with prolonged DOC during hospitalization were screened by clinicians, and the clinicians formed a clinical-consensus diagnosis. Trained professionals used the CRS-R to evaluate the consciousness levels of the enrolled patients repeatedly (≥5 times) within a week. Based on the repeated evaluation results, the enrolled patients with prolonged DOC were divided into unresponsive wakefulness syndrome (UWS), minimally conscious state (MCS), and emergence from MCS (EMCS). Finally, the relationship between the results of the CRS-R and the clinical consensus were analyzed.

**Results:**

In this study, 137 patients with a clinical-consensus diagnosis of prolonged DOC were enrolled. It was found that 24.7% of patients with clinical UWS were actually in MCS after a single CRS-R behavior evaluation, while the repeated CRS-R evaluation results showed that the proportion of misdiagnosis of MCS was 38.2%. A total of 16.7% of EMCS patients were misdiagnosed with clinical MCS, and 1.1% of EMCS patients were misdiagnosed with clinical UWS.

**Conclusions:**

The rate of the misdiagnosis by clinical consensus is still relatively high. Therefore, clinicians should be aware of the importance of the bedside CRS-R behavior assessment and should apply the CRS-R tool in daily procedures.

**Trial registration:**

ClinicalTrials.gov ID: NCT04139239; Registered 24 October 2019 - Retrospectively registered.

## Background

The most severe injuries result in prolonged (i.e., lasting at least 28 days) disorders of consciousness (DOC), including unresponsive wakefulness syndrome (UWS) [1–3] and minimally conscious state (MCS) [4, 5]. Currently, the boundary between UWS and MCS patients has been well-defined [3–5]. The main difference between UWS and MCS is whether there is definite evidence that patients have a certain ability to be aware of themselves and the outside world, which is present only in patients with MCS. Clinical evaluations of the level of consciousness in patients with prolonged DOC have been conducted mainly through bedside spontaneous and stimulating behavioral responses [6–8]. The level of arousal is reflected in the assessment of the patient’s open eye, whereas awareness is mainly assessed based on the patient’s perception of himself or herself and the external environment, i.e., the patient’s non-reflexive behavior under stimulation or the evaluator’s accidental discovery of the patient’s non-reflexive behavior. UWS patients have clear characteristics of awakening without awareness [9]. There are spontaneous or stimulus-induced open-eye reactions, sleep-wake cycles, and spontaneous reflexes (such as grunts and yawns), but there is a complete lack of awareness of oneself or the environment in UWS patients [8, 10]. That is, there is no clear evidence of awareness or directed response to external stimuli; however, the presence of repetitive non-reflexive behavioral responses suggests a transition to the MCS state. MCS patients can generally show some behavioral response characteristics related to consciousness [4, 11]. In these patients, there is weak and fluctuating but definite behavioral evidence of a distinct sense of self or of the environment, such as the ability to visually track objects and the ability to understand verbal information and follow instructions. At the same time, MCS can be further divided into MCS plus (MCS+) and MCS minus (MCS-) subtypes according to the complexity of the behavioral response (such as the presence of language comprehension) [5]. Once patients can communicate functionally or can use functional objects, they are diagnosed with emergence from MCS (EMCS) [12, 13].

Due to the difficulty of performing bedside consciousness assessments of patients with prolonged DOC, the rate of misdiagnosis is very high [14]. Different diagnosis results are crucial for clinical treatment, nursing, and even affect the decision of life termination [7, 15, 16]. For example, the treatment of transcranial direct current stimulation may be more effective in MCS than in UWS patients [17]. MCS is probably associated with a better prognosis than UWS [10]. In addition, Boly et al. found that the perception of pain is preserved in MCS patients, which indicated that these patients may need analgesic treatment [18]. The development and use of the Coma Recovery Scale–Revised (CRS-R) has greatly reduced the rate of clinical misdiagnosis of prolonged DOC [19]. The scale measures the patient’s auditory, visual, motor, oromotor, communication and arousal function to assess the patient’s level of consciousness and is now the most reliable diagnostic tool for patients with prolonged DOC [20]. Previous studies have shown that a single CRS-R assessment can identify 41% MCS patients who had been misdiagnosed with UWS based on clinical consensus [21]. In addition, recent studies have shown similar results, with a misdiagnosis rate of 35.3% for clinical consensus [22]. To date, several versions of the CRS-R scale have been developed and validated [23–27]; however, due to the influence of patients’ awakening or consciousness fluctuations, movement defects, aphasia, and other problems [28–30], a single standard CRS-R behavior evaluation still leads to a nonzero rate of misdiagnosis. Therefore, repeated behavior scale evaluations [31] and personalized item selection [32, 33] of the neurobehavioral-assessment instrument have been for consciousness evaluations of clinical patients in order to improve the reliability of diagnosis.

In recent years, the concept of prolonged DOC has been clinically wide spread [10, 34]; however, the CRS-R is still not often used in clinical practice but is mostly used by clinical psychologists and specialized researchers. In this study, the difference between clinicians’ diagnoses and the diagnosis of a single CRS-R assessment was compared, and the proportion of misdiagnosis by clinical consensus compared with the assessment results of a repeated CRS-R was analyzed.

## Methods

### Patients

Patients with prolonged brain injuries admitted by the neurology department and the neurological rehabilitation department were primarily enrolled. The inclusion criteria were as follows: (1) at least 16 years old, (2) 28 or more days elapsed after onset, and (3) no neuromuscular blockers or sedatives were used within 72 h of enrollment. The exclusion criteria were: (1) being in a coma, (2) presence of functional disorders caused by progressive mental diseases, (3) persistent seizures, and (4) unstable vital signs.

This study protocol was approved by the Ethics Committee of Hangzhou Normal University. Written informed consent was obtained from the guardians/next of kin of the patients who participated in this study.

### Data collection

Patients with prolonged DOC during hospitalization were screened by clinicians, and the clinicians in charge determined the patients’ consciousness according to behavior during hospitalization (including the Glasgow Coma Scale assessment and other physical examinations) and their own clinical experience to form a clinical-consensus diagnosis. Patients included those with UWS with no consciousness and MCS with minimal consciousness. Subsequently, at least two experienced clinical psychologists used the CRS-R to evaluate the neurological behavior of the enrolled patients in five or more assessments over the following week (once or twice a day). Each patient’s diagnostic results and special behaviors were recorded. From the repeated evaluation results, the highest scoring diagnosis was selected as the final bedside behavior diagnosis. All patients with prolonged DOC were divided into UWS, MCS-, MCS+, and EMCS groups.

### Statistical analysis

A statistical evaluation was performed for all demographic information. Number, percentage, median, and range were produced for categorical variables, and means and standard deviations (SD) were calculated for age, time since onset, and the score on the CRS-R scale.

A chi-square test (and Fisher exact test when necessary) was used to test for group differences in demographic characteristics (i.e., gender, etiology, age and injury time) on the proportion of misdiagnosis. A Mann–Whitney U test (Wilcoxon rank-sum test) was used to analyze the difference between the scores of the first and final diagnoses. The statistical significance was set at *p* < 0.05.

## Results

A total of 137 patients with prolonged DOC selected by their clinicians were included in the study, which took place between July 2017 and October 2019. Baseline patient characteristics (numbers, percentage, median, and range or mean ± SD) are provided for all included research individuals (Table 1). There were 40 female patients (29.2%) and 97 male patients (70.8%). Sixty-nine patients had suffered traumatic brain injuries (TBI, caused by a violent blow or jolt to the head or body) (50.4%), and 61 patients had suffered cerebrovascular accidents (CVA, hemorrhage caused, infarction and subarachnoid hemorrhage) (44.5%), and 7 patients had suffered anoxic brain injuries (ABI) (5.1%). The mean age was 51.88 ± 13.93 years (range 19 to 84 years). The mean time since onset was 5.58 ± 4.32 months (range 1 to 22 months), and 44 patients suffered from permanent DOC (32.1%).

**Table 1:** Demographic characteristics and clinical data of patients with prolonged DOC

Characteristics/Variables	*n*	%	Mean ± SD	Median (range)
Sex				
Male	97	70.8		
Female	40	29.2		
Etiology				
TBI	69	50.4		
CVA	61	44.5		
ABI	7	5.1		
Age (years)	137		51.88 ± 13.93	52 (19–84)
16–44	37		34.41 ± 6.9	35 (19–44)
45–59	57		51.3 ± 4.44	51 (45–59)
≥ 60	43		67.7 ± 5.95	66 (60–84)
Time post-onset (m)				
Whole sample	137		5.58 ± 4.32	4 (1–22)
Non-permanent	93	67.9	3.92 ± 2.45	3 (1–11.5)
Permanent	44	32.1	9.1 ± 5.23	7 (4–22)
CRS-R scores				
Single assessment	137		7.93 ± 4.3	7 (2–24)
Repeated assessment	137		8.87 ± 4.32	8 (2–24)

*SD* Standard deviation, *DOC* Disorders of consciousness, *TBI* Traumatic brain injury, *CVA* Cerebrovascular accident, *ABI* Anoxic brain injury, *CRS-R* Coma Recovery Scale-Revised, *n* numbers, *m* months

*Permanent* = three months after postinjury (non-traumatic), 12 months after postinjury (traumatic)

The mean score of the single CRS-R assessment was 7.93 ± 4.3 points (range 2 to 24), and the mean score of the final CRS-R assessment was 8.87 ± 4.32 points (range 2 to 24), which was higher than the single assessment score (*U* = 7827.5, *p* = 0.02).

**Fig. 1:**
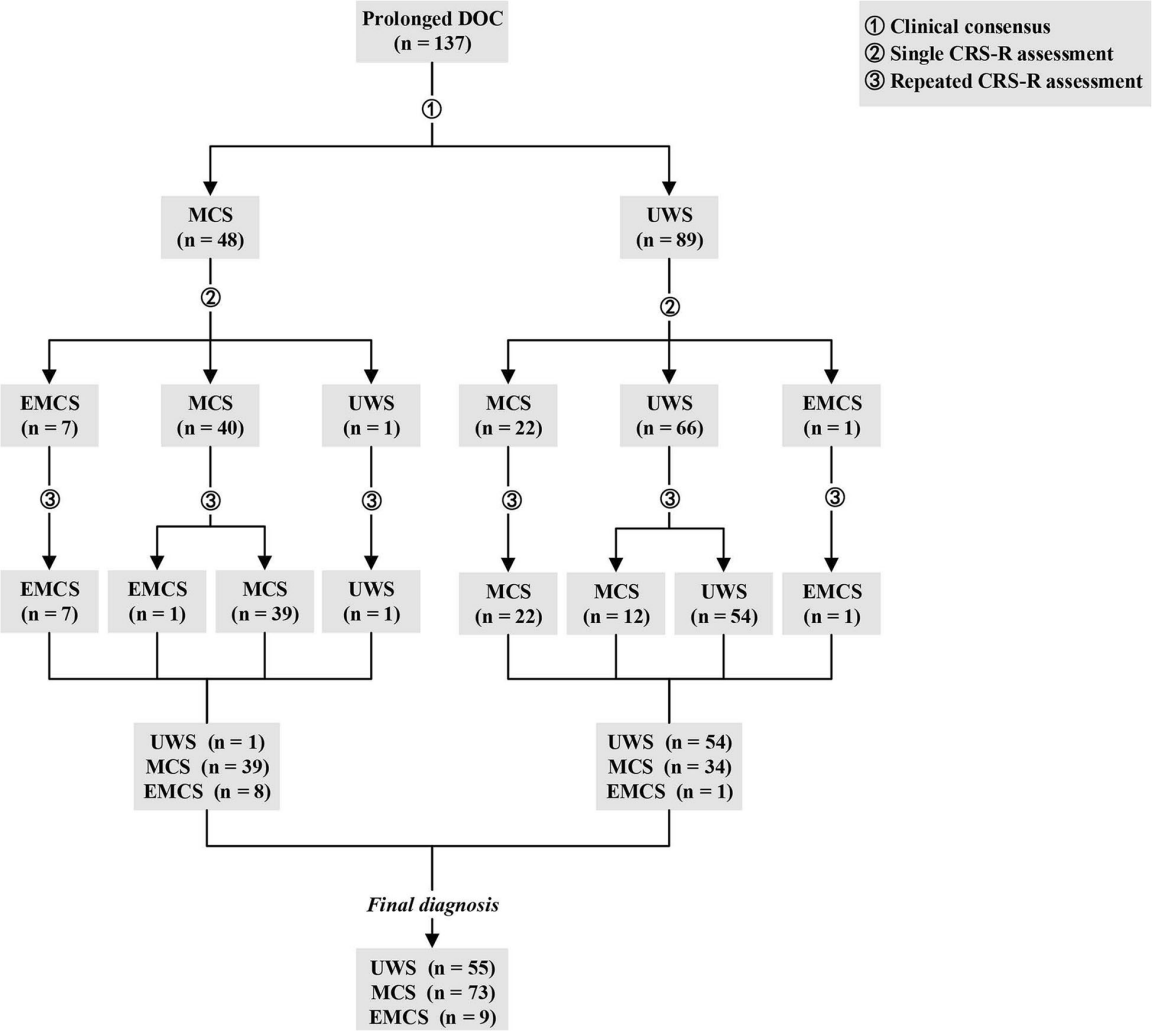
Flowchart of participants through the study. Of the 137 patients with prolonged DOC enrolled, 48 were diagnosed by clinical consensus as MCS and 89 as UWS. After a single CRS-R evaluation, 62 were diagnosed with MCS, 8 with EMCS, and 67 with UWS. After repeated CRS-R evaluations, 73 were diagnosed with MCS, 9 with EMCS, and 55 with UWS. *DOC* = disorders of consciousness; *UWS* = unresponsive wakefulness syndrome; *MCS* = minimally conscious state; *EMCS* = emergence from minimally conscious state; *n* = numbers

Figure 1 shows the diagnostic process and results for all patients with prolonged DOC. Of the 137 patients with acquired brain injury enrolled, 48 were diagnosed by clinical consensus as MCS and 89 as UWS. After a single CRS-R evaluation, 62 were diagnosed with MCS, 8 with EMCS, and 67 with UWS. After repeated CRS-R evaluations, 73 were diagnosed with MCS, 9 with EMCS, and 55 with UWS.

It was also found that after a single CRS-R evaluation, 7 of the 48 patients with a diagnosis of MCS based on clinical consensus were diagnosed with EMCS (7/48 = 14.6%), 40 with MCS (40/48 = 83.3%), and 1 with UWS (1/48 = 2.1%) (Table 2). After repeated CRS-R evaluations, 1 of the 40 patients diagnosed with MCS based on a single CRS-R assessment was diagnosed with EMCS (1/40 = 2.5%) and 39 with MCS (39/40 = 97.5%, with 2 diagnoses revised from MCS- to MCS+). The diagnosis of 1 UWS was still UWS, and 7 EMCS were still diagnosed with EMCS. Among the 89 patients with clinical UWS, the diagnosis of 22 patients was improved to MCS (22/89 = 24.7%) after a single CRS-R evaluation. One was improved to EMCS (1/89 = 1.1%), and 66 maintained the diagnosis of UWS (66/89 = 74.2%). After repeated CRS-R evaluations, 12 of the 66 patients diagnosed with UWS based on a single CRS-R assessment were diagnosed with MCS (12/66 = 18.2%), while 54 were diagnosed with UWS (54/66 = 81.8%), while 22 MCS were still diagnosed with MCS (22/22 = 100%, but 1 diagnosis was improved from MCS- to MCS+), 1 EMCS was still diagnosed with EMCS.

**Table 2:** Numbers of misdiagnosis in relation to demographic profiles of patients with prolonged DOC in different diagnosis settings

	Clinical consensus		Single assessment		Repeated assessment	
	UWS, *n*	MCS, *n*	MCS, *n* (%) ^a^	EMCS, *n* (%) ^b^	MCS, *n* (%) ^a^	EMCS, *n* (%) ^b^
Sex						
Male	65	32	18 (27.7)	4 (12.5)	28 (43.1)	4 (12.5)
Female	24	16	4 (16.7)	3(18.8)	6 (25)	4 (25)
*p* value			> 0.05	> 0.05	> 0.05	> 0.05
Etiology						
TBI	44	25	11 (25)	4 (16)	17 (38.6)	5 (20)
CVA	38	23	11 (28.9)	3 (13.0)	17 43.6)	3 (13.0)
ABI	7	0	0	0	0	0
*p* value			> 0.05	> 0.05	> 0.05	> 0.05
Age (years)						
16–44	27	10	6 (22.2)	1 (10)	11 (41)	1 (10)
45–59	37	20	12 (32.4)	4 (20)	16 (43.2)	5 (25)
≥ 60	25	18	4 (16)	2 (11.1)	7 (28)	2 (11.1)
*p* value			> 0.05	> 0.05	> 0.05	> 0.05
Time post-onset						
Permanent	27	17	6 (22.2)	1 (5.9)	9 (33.3)	1 (5.9)
Non-permanent	62	31	16 (25.8)	6 (19.4)	25 (40.3)	7 (22.6)
*p* value			> 0.05	> 0.05	> 0.05	> 0.05
Total	89	48	22 (24.7)	7 (14.6)	34 (38.2)	8 (16.7)

^a^Numbers of MCS patients were misdiagnosed as UWS; ^b^Numbers of EMCS patients were misdiagnosed as MCS

*DOC* Disorders of consciousness, *UWS* Unresponsive wakefulness syndrome, *MCS* Minimally conscious state, *EMCS* Emergence from minimally conscious state, *TBI* Traumatic brain injury, *CVA* Cerebrovascular accident, *ABI* Anoxic brain injury, *χ2* Chi-square, *n* numbers

Overall, 24.7% of MCS patients were misdiagnosed with UWS based on clinical consensus after single evaluations (22/89). The proportion of EMCS being misdiagnosed with MCS was 14.6% (7/48 = 14.6%). After repeated evaluations, 38.2% of MCS patients were misdiagnosed with UWS based on clinical consensus (34/89). The proportion of EMCS misdiagnosed with MCS was 16.7% (8/48 = 16.7%) (Table 2). Table 2 also shows the rate of misdiagnosis by clinical consensus for different demographic variables, including frequency and proportion of misdiagnosis. It was found that there was no significant difference in the proportion of misdiagnosis among different genders, etiologies, age groups, and time since onset (*p* > 0.05).

**Fig. 2:**
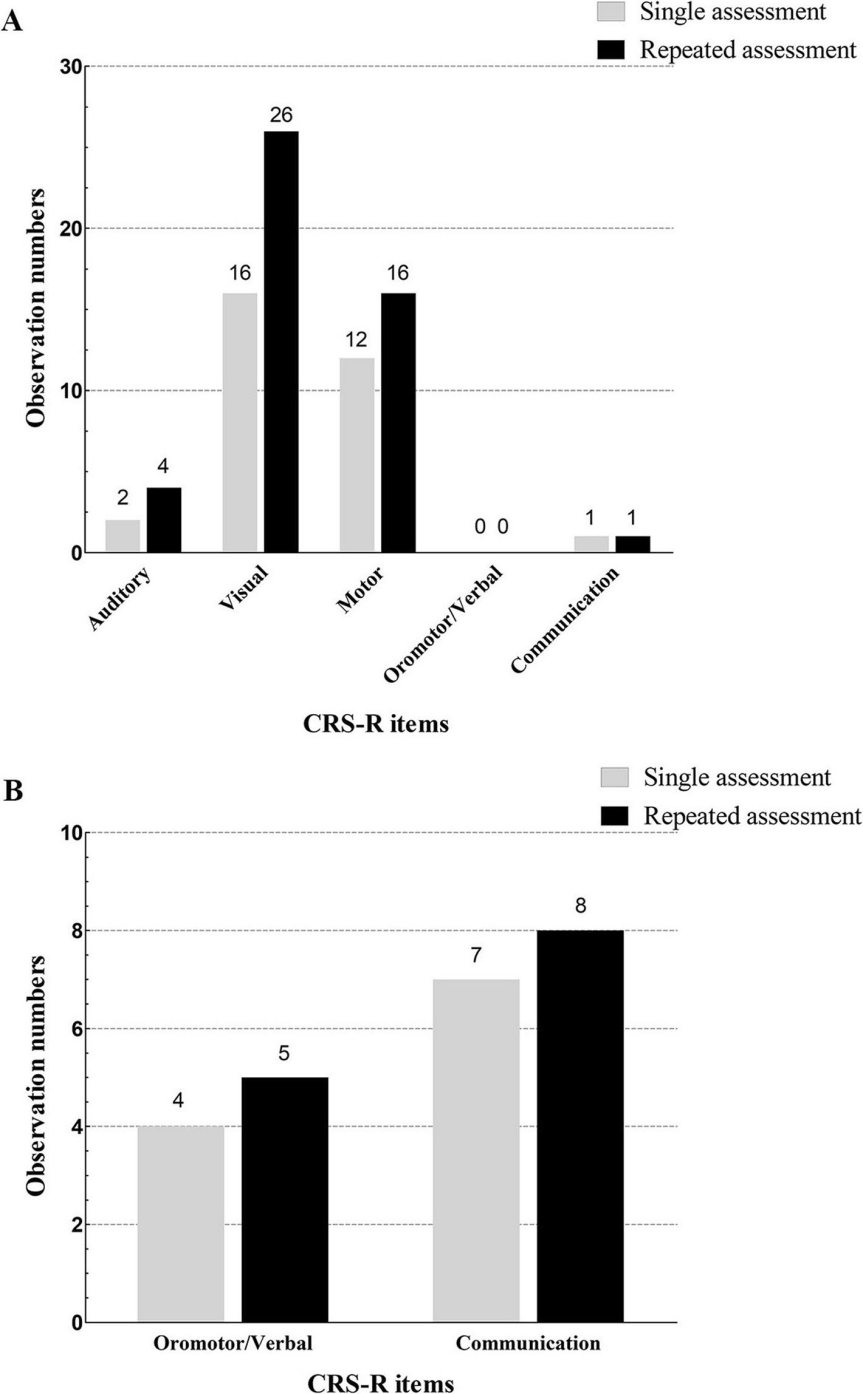
The number of CRS-R subscales representing signs of consciousness when diagnosed with MCS or EMCS after a single CRS-R assessment and after repeated CRS-R assessments. **a**. In these terms, Auditory = 3–4 *OR* Visual = 2–5 *OR* Motor = 3–5 *OR* Oromotor/Verbal = 3 *OR* Communication = 1, indicating that the patient has signs of consciousness and is diagnosed as MCS. Of the patients with a clinical consensus diagnosis of UWS, 22 were diagnosed with MCS after a single CRS-R assessment. After repeated CRS-R assessments, 34 patients were diagnosed with MCS. **b**. In these terms, Motor = 6 *OR* Communication = 2, indicating that the patient has signs of full consciousness and is diagnosed as EMCS. Of the patients with a clinical consensus diagnosis of MCS and UWS, 8 were diagnosed with EMCS after a single CRS-R assessment. After repeated CRS-R assessments, 9 patients were diagnosed with EMCS. *CRS-R* = Coma Recovery Scale-Revised; *MCS* = minimally conscious state; *EMCS* = emergence from minimally conscious state

Figure 2 shows the number of patients with CRS-R subscales representing signs of consciousness when diagnosed with MCS or EMCS after a single assessment and after repeated assessment. After a single CRS-R assessment, among 22 patients diagnosed with MCS, 2 patients showed a sign of consciousness on the auditory subscale (9.1%), 16 patients on the visual subscale (72.7%), 12 patients on the motor subscale (54.5%), 0 patients on the oromotor/verbal subscale (0%), and 1 patient on the communication subscale (4.5%). After repeated CRS-R assessments of 34 patients with a diagnosis of MCS, 4 patients showed signs of consciousness on the auditory subscale (11.8%), 26 on the visual subscale (76.5%), 16 on the motor subscale (47.1%), 0 on the oromotor/verbal subscale (0%), 1 on the communication subscale (2.9%). Of the 7 patients diagnosed with EMCS after a single assessment, 4 (57.1%) scored on the motor subscale, and 7 (100%) scored on the communication subscale. After repeated evaluations, 5 (55.6%) of the 9 patients diagnosed with EMCS scored on the motor subscale, and 8 (88.9%) scored on the communication subscale.

## Discussion

The main objective of this study was to investigate the misdiagnosis rates of clinical consensus compared to those of repeated behavior-scale assessments. After the single CRS-R behavior evaluation, it was found that the proportion of misdiagnosis of clinical MCS was 24.7%, while the repeated CRS-R evaluation results showed that the proportion of misdiagnosis of clinical MCS was 38.2%. A total of 16.7% of EMCS patients was misdiagnosed with MCS, and 1.1% of EMCS patients was misdiagnosed with UWS.

For the evaluation of the consciousness level of patients with prolonged DOC, many previous studies had compared the diagnostic results of the standard CRS-R scale with other scales, and it was been found that the CRS-R scale had the highest sensitivity in detecting the consciousness of patients with MCS [35, 36]. When the CRS-R scale was used, it was found that many patients with a clinical-consensus diagnosis of unconscious actually remained minimally conscious. Schnakers et al. found that 41% of patients with a clinical-consensus diagnosis of UWS was actually found to suffer from MCS after an evaluation using the standard CRS-R behavior scale, whereas the clinical consensus was that 10% of patients with MCS were actually higher conscious EMCS (fully conscious) [21]. A recent study on repeated CRS-R behavior assessments showed that the clinical consensus still had a 33% misdiagnosis rate when diagnosing MCS patients [22]. This also supports the results of the current study. It was found that repeated behavioral assessments could identify 38.2% MCS patients. Moreover, the proportion of misdiagnosis of EMCS with full consciousness was 16.7%. When the evaluation results of the single CRS-R scale were compared with the clinical consensus, it was found that 24.7% of patients were misdiagnosed with MCS by clinical consensus, which was significantly lower than the 41% found in previous studies on the proportion of misdiagnosis; however, the 14.6% misdiagnosis rate of EMCS was similar to the 10% rate in a previous study [21]. With the continuous progress in the field of prolonged DOC, clinicians have gained a deeper understanding of this concept. This may be the reason for the significant difference in misdiagnosis rates over the past 10 years. In addition, the difference between the results of single assessments and repeated assessments emphasizes that the fluctuations of patients’ responsiveness have an effect on neuro-behavioral assessments, and also emphasizes the importance of repeated assessments in clinical diagnosis.

The degree to which the patient’s demographic factors lead to clinical misdiagnosis was analyzed, and it was found that differences in gender, etiology, age groups and postinjury time were not factors in clinical-consensus misdiagnosis. It is highly likely that clinical workers are highly dependent on the patients’ bedside behaviors in the patients’ daily management and may not be using systematic and standardized behavioral-assessment tools to diagnose awareness. In addition, it was found that the Glasgow Coma Scale (GCS) was widely used for almost all patients admitted to the hospital, while a previous study showed that that scale was not appropriate for assessing a patient’s level of consciousness [23]. Unlike the GCS scale, the CRS-R scale has very clear MCS diagnostic criteria, and the evaluation of consciousness from various angles can be used to more sensitively diagnose the consciousness level of patients, which greatly reduces the misdiagnosis of patients with prolonged DOC. Therefore, the use of standardized CRS-R assessment tools is particularly important for the detection of clinical patients’ level of consciousness and patient management.

During the implementation of the standardized CRS-R scale, many studies found that the standard CRS-R scale still lead to some misdiagnoses. Cheng and Gosseries et al. found that the patient’s name was more suitable stimulus for the detection of auditory localization than other sound stimuli [37]. Vanhaudenhuys et al. also found that the best way to check visual pursuit in MCS patients was to use a moving mirror rather than a moving person or object [38, 39]. Therefore, the application of personally related visual and auditory stimulation can better reduce the proportion of misdiagnosis of patients compared with natural stimulation [33]. In addition, when the CRS-R was used to evaluate the use of functional objects for MCS patients, the use of personalized objects more frequently elicited responses from patients, thereby identifying misdiagnosed EMCS [32]. For this study, repeated CRS-R behavior assessments were employed, during which family members or caregivers were asked about patients’ items of interest. To better elicit the patients’ responses, a variety of different stimuli were selected according to the patients’ performance during the evaluation process, namely natural stimuli and personally related stimuli. It was found that when patients were diagnosed with MCS based on the first behavior evaluation, most showed signs of consciousness on the visual (72.7%) and motor (54.5%) subscales, and few showed signs of consciousness on the auditory (9.1%) and communication (4.5%) subscales. After repeated evaluations, 10 patients showed signs of consciousness on the visual subscale, 4 patients on the motor subscale, and 2 patients in the auditory subscale. This was most likely due to fluctuations in the patients’ levels of arousal or consciousness and due to the use of personally associated stimuli.

It was also found that, for the vast majority of patients diagnosed with MCS, the items eliciting signs of consciousness were mainly related to the visual subscale (visual pursuit and visual fixation), the motor subscale (automatic motor response and localization to noxious stimulation), and the auditory subscale (reproducible movement to command). These results were confirmed by a previous study [40], but with the difference that the most sensitive item in the present study was the visual subscale, while the most sensitive item in the previous study was the reproducible movement to command items on the auditory subscale.

Based on these results, it was found that the clinical consensus had a higher proportion of misdiagnosis, especially compared to repeated CRS-R scales. This highlights the importance of the CRS-R scale in the assessment of patient consciousness. It is suggested that, for patient daily management, clinicians should at least evaluate visual pursuit and visual fixation for the visual subscale, automatic motor response and localization to noxious stimulation for the motor subscale, and reproducible movement to command for the auditory subscale when assessing patients’ levels of consciousness. This can greatly reduce misdiagnosis, although, for patients with prolonged DOC, bedside neurobehavioral assessment has some limitations, and neuroimaging is an important method for the diagnosis of consciousness [22]; however, a behavioral assessment is still the most direct and portable method and should be promoted in clinical practice.

The limitation of this study was that no neuroimaging methods were used to evaluate the enrolled patients with prolonged DOC. Because the CRS-R scale still produces some false negatives, in the future, behavioral assessments combined with neuroimaging should be used to truly understand misdiagnosis by clinical consensus.

## Conclusions

Although many studies have emphasized the importance of diagnosis, this study showed that the current rate of misdiagnosis by clinical consensus is still relatively high. Misdiagnosis greatly affects the clinical management of patients with prolonged DOC. Therefore, clinicians should be aware of the importance of bedside CRS-R behavior assessments and should apply the CRS-R scale to their daily procedures. Choosing stimuli that are relevant to the patient and evaluating them on a standard scale can better identify the patient’s covert consciousness. For a rapid evaluation of patients, visual pursuit and visual fixation for the visual subscale, automatic motor response and localization to noxious stimulation for the motor subscale, and reproducible movement to command for the auditory subscale are recommended.

## Data Availability

The datasets used and/or analyzed during the current study are available from the corresponding author on reasonable request.

## Abbreviations

DOC: Disorders of Consciousness
UWS: Unresponsive Wakefulness Syndrome
MCS: Minimally Conscious State
MCS+: Minimally Conscious State Plus
MCS-: Minimally Conscious State Minus
EMCS: Emergence from Minimally Conscious State
CRS-R: Coma Recovery Scale-Revised
TBI: Traumatic Brain Injury
CVA: Cerebrovascular Accident
ABI: Anoxic Brain Injury
